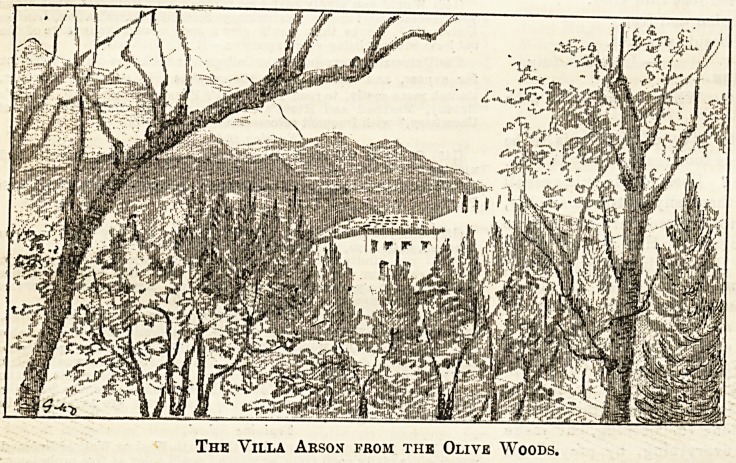# "The Hospital" Nursing Mirror

**Published:** 1899-01-07

**Authors:** 


### "The Hospital" Nursing Mirror.

Being the Nursing Section of "The Hospital."

[Contributions for this Section of "The Hospital" should be addressed to the Editor, The Hospital, 28 & 29, Southampton Street, Strand, London, W.C., and should have the word "Nursing" plainly written in left-hand top corner of the envelope.]

### News from the Nursing World.

### NEW YEAR'S HONOURS.

We rejoice greatly at seeing the names of nine nurses in the Queen's list of New Year's honours, and congratulate them heartily upon it. Their names are: Miss Lilian M. Robinson, Bombay; Miss Maud B. Kendall, Bombay; Miss Jane E. Wheatley, Poona; Miss Emma A. Moles, Poona; Miss J. E. Blair Hitchman, Sholapur; Miss Marion Hale, Cutch; Miss Harriet J. Horne, Karachi; Sister Heriberta, Karachi; Mrs. Dyson, Surat. Plague nursing, the work which they were called upon to do, is arduous, dangerous, and disagreeable, and its recognition by Her Majesty cannot fail to be gratifying, not only to themselves but to all women. The honour bestowed upon them is the same that was granted to the Inniskea nurses, that is, they are appointed Honorary Serving Sisters of the Order of the Hospital of St. John of Jerusalem in England. They will be entitled to wear the badge of the Order.

### THE GREEK QUEEN AND THE WAR NURSES.

On the 2nd inst. the Queen of Greece distributed commemorative crosses to 80 of the nurses, trained and untrained, who had served in the Red Cross Hospital during the war. Amongst the recipients were the Princess Marie, the Crown Princess Sophie of Greece, and the English nurses who took part in attending the soldiers. Much attention is paid by the Greek Royal Family in organising and establishing hospitals in that country. English methods have been studied for the purpose, and English nurses are engaged to superintend their inauguration.

### THE NURSING OF UNIVERSITY COLLEGE HOSPITAL.

University College Hospital is the only hospital in London nursed by the sisters of an Anglican Community. For 34 years the All Saints' Sisters, under Sister Cecelia, the Superior, have been entirely responsible for it. The hospital authorities have allowed them a certain sum for this purpose, which has been spent in the payment of nurses' salaries, probationers, and in their maintenance, but the services of the sisters have been free. The last years have been a period of great stress; the encroachment of the new building upon the old, the outbreak of typhoid amongst the nurses, and the consequent rearrangement of the nurses' accommodation entailed much anxiety and work. Then the Sister Superior's health necessitated a long rest, which she spent in a visit to India, returning to her duties well again. A more far-reaching change will take place in June next, when the staff of 18 sisters will withdraw from the superintendence of the nursing. Under the conditions involved by the building of a new hospital and nurses' home the whole system of nursing needed reorganisation. By the mutual consent, therefore, of the sisters and the committee, between whom the relationship is, as it has always been, most cordial, it has been deemed best to place it in other hands, whilst, in deference to the wishes of the younger medical men, the example of other hospitals will be followed, and the new matron and sisters will be laywomen.

### THE QUEEN'S NURSES OF SCOTLAND.

On December 26th the annual meeting of the Scottish branch of Queen Victoria's Jubilee Institute for Nurses was held at the Home in Edinburgh. We noted the rearrangement of the work in consequence of the increased number of the staff some little time ago, and therefore confine our remarks to matters financial. When the Duke of Westminster inaugurated the "Queen's Commemoration Fund" in honour of the sixtieth year of her reign, for the purpose of augmenting the capital of the great scheme of national district nursing, it was arranged that one-fifth of all moneys collected should be handed over to the Scottish branch. When, however, it was found that Scotland gave more than this amount the whole of her contributions—a sum of £12,193—was handed over to the Council of that branch. Of this sum £10,000 has been invested, and will be held as a reserve fund, of which the interest will be used for current expenses, and the capital retained for extraordinary calls of a special nature. The possession of this capital does not in the least detract from the necessity of continued support from the public. The work continues to grow, the demand for nurses increases, and the expenses of training nurses are heavier, and last year the society spent £705 more than its income.

### "THE HOSPITAL."

Some little time ago a subscriber kindly offered to send her copy of The Hospital on to "a nurse too poor to buy it for herself." It was not without some misgivings that we inserted her offer, knowing that there were many nurses who would be glad to receive it on such terms, and dreading the avalanche of letters that would surely follow it. After the first few answers were forwarded, the rest were set aside until the press of Christmas work was over, and now two more nurses have sent saying they will gladly send on their copies to poorer sisters. It seems a pity that such kindly co-operation should not be encouraged, but, at the same time, it is quite impossible for us to undertake the distribution.

### THE MEASURE OF SUCCESS.

Sister Dorothy, the hon. matron of the Princess Christian Cottage Hospital, Freetown, Sierra Leone, has just returned to her duties after a visit home. Before leaving England she added her valuable testimony to the success of trained nursing in saving life in that most unhealthy region. Since 1892, when this little hospital of twelve beds, with one ward for Europeans and another for natives, was founded, she has had practical experience on which to found her opinion, and it is therefore one that must help forward the movement now afoot to establish sanatoria and nursing centres in our colonies. But whilst the "success" attendant upon skilled nursing is rightlystrongly brought forward, the fact that success is no standard by which to measure the "need" should not be lost sight of. The duty of succouring and soothing those who will probably die is as imperative as that of helping those who are on their way to convalescence. This fact should be more widely borne in mind, for the duties of a very large number of our nurses, especially those employed in poor law institutions, are with the chronic complaints of age, to which death is the only and natural end. None of the brilliant success in the usual acceptance of the term attends their labours; honours are not showered upon them by the great, yet surely the need of their work is as imperative, the faithful performance of it as holy as any, although their success lies only in making easy the deathbed of perhaps a social failure.

### A NURSE'S AGE.

Considerable amusement has been caused by a nurse applying for the post of charge nurse at the Hampstead Workhouse being very uncertain as to her age. It is one of the ironies of the Superannuation Act that it makes it well-nigh impossible for a nurse getting on in years to obtain employment; for if she break down permanently in health or manage to pass the magic limit of sixty years of age when in the employ of the guardians, she is entitled to a superannuation allowance. The Act, therefore, instead of securing the old nurse's maintenance for life, debars her earning her livelihood during a considerable number of her waning years, and makes it profitable to the Guardians to get rid of her before the age limit is reached. It is the opposite of amusing, therefore, when under these circumstances an elderly nurse "forgets" how old she really is.

### UNMARRIED MATERNITY.

The suffering and shame that are the portion of the unmarried mother and the crimes resultant therefrom are attracting considerable attention at the present moment in consequence of the numerous unhappy cases now, and lately, in our courts of justice. On Friday last the subscribers to the Liverpool Ladies' Charity and Lying-In Hospital held a special meeting, and by 45 votes to 31 rescinded a rule that has been in force since the foundation of the charity a hundred years ago, restricting its benefits to married women. The new resolution ran as follows: "That single women, in exceptional circumstances, who, after careful investigation by the Ladies' Committee, are found to be deserving objects of charity, shall be eligible for admission into the hospital for their first confinement." The question, of course, is not shall good women stand aloof from the trials of their unfortunate sisters, but is it wise to admit the unfortunate into the fellowship of the benefits founded for the respectable poor? Little is to be gained, if by opening the door to a class whose sufferings command sympathy, if not respect, those to whom respect, as well as sympathy, is due are sent away. The Liverpool Ladies' Committee will soon be in a position to judge from experience if this be so or not, and if it be so some means for helping both must be devised.

### ST. LAWRENCE'S CATHOLIC HOME.

We have before us the seventh report of the St. Lawrence's Catholic Home, Dublin. This home occupies the unique position of being the one training school for Roman Catholic district nurses. It is in affiliation with Queen Victoria's Jubilee Institute for Nurses, and it gives the nurses sent by the Institute six months' instruction in district work. The number of nurses on the staff is six, and a large amount of good work is undoubtedly done by it for the indigent sick of Dublin. The Countess of Cadogan has countenanced the home, but subscriptions are much needed if the committee are to continue its usefulness unhampered by debt.

### NURSING AT SHEFFIELD.

The ninth annual report of the St. George's Nurses Home is not very satisfactory. Unfortunately, owing to sickness of the staff, and also to that staff being reduced by one nurse, the number of visits has decreased considerably. It is always disheartening to workers to feel themselves unable to cope with their work, and this is especially so when the sick claim their care. This year 1,123 cases were nursed, involving 20,172 visits. The home has many kind friends who have added greatly to the comfort of the nurses in many ways. One supplies a cab for the conveyance of nurses to and from their district, another tram tickets, but, so far, nothing has been said about bicycles.

### AN AGGRIEVED NURSE.

Quite a storm has been created in the councils of the Bandon (Ireland) Guardians, in consequence of their refusal to appoint a nurse lately in their employ to a vacant berth in their hospital. The aggrieved lady wrote to the Local Government Board, asking them to investigate the matter, and sent the medical officer a lawyer's letter on account of his testimony before the Board as to her suitability for the post. The rights and wrongs of this lady's case are matters of little import, save only that they show the amazing misconception some people have of their own position, and the rights attached to it. In the first place, the Guardians have an unqualified right to select or reject any individual candidate for their employment, and to give no account or reason for their decisions to anyone; that is, provided they keep within the rulings of the Local Government Board. In the second, a communication from a medical officer to his employers concerning his proper duty is privileged. And, lastly, a nurse or any other officer who runs a tilt against the powers that be, must expect to be worsted in the fray.

### SHORT ITEMS.

The Guy's nurses who have hitherto been housed in the old "Mary" ward have been transferred to the temporary nursing home lately prepared for them. Their old quarters will be used by the patients of each surgical ward in turn whilst these wards are thoroughly cleaned. "Mary" will eventually be used as an additional medical ward.—The District Trained Nursing Society, Adelaide, N.S.W., has lost its able and popular president, Dr. Allan Campbell, through death. He had been associated with the society since its formation, and it has been owing to his influence that the society has progressed so rapidly.—The public memorial to be given to the Dominican Sisters upon their relinquishing the nursing of the Bulawayo Hospital by the residents in Rhodesia is to take the form of a Home. Each Sister will receive in addition a silver cross and a framed testimonial.—The supplementary bazaar for providing the Argyllshire County Nursing Association with the money was opened on December 30th by Captain Hector Macneal at Campbeltown. — The engagement of Miss Gertrude Hick, matron of the General Hospital, Kettering, to Dr. Josiah Oldfield, of Harley Street, is announced.

### Antiseptics for Nurses.

By a Medical Woman.

### XXIII.—APPARATUS FOR DISINFECTION BY HEAT.

A modification of the Washington Lyon's apparatus is one by Alliott and Paton, of which the chief feature is the addition of a vacuum-producing apparatus, which is so attached that the air is exhausted not only from the inner chamber, but also from the interstices of the articles to be disinfected, which greatly facilitates the penetration of the steam. Moreover, when the process of disinfection is completed, the steam can be exhausted by an air-pump, so that no deposit of moisture takes place when the doors are opened, and the articles are consequently quite dry. This same apparatus may also be used as a hot-air or as a dry-air disinfector.

The Bradford Patent Disinfecting Apparatus consists of two parts, one a container, and the other the heating apparatus. The container is a large rectangular iron box, covered with a non-conducting composition, and open below. It is suspended by chains and counterpoises from pillars, something like a gas-holder. The roof has an opening for ventilation, and attached to the side is a thermometer having its bulb projecting into the interior, whilst the stem is outside, and thus the temperature in the interior can be easily observed. The heating apparatus consists of three compartments placed side by side, the central one of which contains the fire waggon. The best fuel for use in this apparatus is peat, but coal, coke, or charcoal can all equally be used. The fire is in a waggon which may be drawn in or out, and the fire chamber containing this waggon has a roof formed of hollow iron bars, open at the two ends, and of triangular form; this chamber also has a flue at one end and a door at the other. The two chambers on either side are for the purposes of ventilation, and communicate with the fire chamber by means of side valves. The articles to be disinfected are arranged on an iron rack which stands over the fire chamber. The container rests on a layer of sand, and a large shallow vessel, filled with water, is placed at the bottom over the fire chamber, so as to keep the air in the chamber moist. The whole apparatus can be run on wheels, so that it may be moved easily from one place to another.

Another apparatus which somewhat resembles that of Washington Lyon is that manufactured by Messrs. Goddard, Massey, and Warner. It is rectangular in shape and large enough to undertake the disinfection of articles for a large district. It is entirely surrounded by a double jacket, and the doors are hollow, and by means of a jointed pipe are connected with the casing, so that they also are double-jacketed. The space, about 4 inches, between the two jackets is filled, the lower half with water, and in the upper half, as well as in the door, the steam circulates; thus a separate boiler is dispensed with, as the boiler is formed by the lower part of the jacket, underneath which is placed the furnace to heat the water, and from the time of lighting it takes less than an hour to get up steam. The pressure in the boiler and jacket is consequently the same, and usually about 20 lb., whilst the temperature of the steam is 240 deg. F.; and on the top is placed a safety-valve weighted to 20 lb. pressure. Steam is admitted into the chamber from the jacket through a screw-down valve, and it is withdrawn through a similar valve by a steam exhaust pipe, discharging into the chimney. Two pressure gauges over the door mark the pressure and temperature in the jacket and chamber respectively. A supply of hot air is laid on through a channel running from the back along the side of the furnace, and discharging through a screw-down valve into the chamber. The pipe is also furnished with a thermometer to mark the temperature of the incoming air, which is regulated by a cold-air supply tap attached. The hot-air and the steam-inlet pipes are carried up to the top of the chamber, and the exhaust outlet-pipe to the bottom. The apparatus is worked as follows: The steam being raised in the jacket to 20 lb. pressure, the chamber is filled with the articles to be disinfected and the doors are closely shut and clamped. The exhaust and hot-air valves are opened, the cooler air is withdrawn from the bottom of the chamber, and displaced from above by a current of hot air. This thoroughly dries the contained articles, displacing the cooler air from their interstices and preventing condensation of steam within them. The hot-air valve is then closed and the steam-inlet valve opened. This discharges steam at the top of the chamber and causes a downward current towards the exhaust, so that the effect of current steam is obtained, by which means the hot air is more or less completely driven out. This riddance of air permits of the full and rapid effects of the steam alone, otherwise the disinfection would be delayed by the admixture of air and steam. The exhaust valve is then closed and the steam pressure allowed to rise to the required degree, the maximum being 20 lb., and the same pressure is now marked by both the gauges.

The Nottingham Self-regulating Disinfecting Apparatus was invented by Dr. Ransome, and consists of a cubical iron chamber cased in wood, with an intervening layer of felt, and access to the interior is obtained by double doors. The furnace, placed at the side of the chamber and on a lower level, consists of a ring of atmospheric gas burners, enclosed in an iron tube. The heated air, containing also the products of combustion, passes along a horizontal flue and enters the chamber at the bottom, which is perforated by a number of holes so that the hot air may be evenly distributed. In the horizontal flue are fixed the bulbs of a thermometer and of a self-acting mercurial regulator; through the latter of these the gas supply to the burners can be made to pass, and it is so constructed that, as the temperature of the apparatus rises, the mercury expands and encroaches upon the slit through which the gas passes, and thus gradually cuts off the supply. At the top of the chamber there is an outlet flue which is controlled by a valve and also furnished with a thermometer. In connection with the outlet is an arrangement designed to extinguish the fire. When the temperature at the outlet exceeds 300 deg. F. a link of fusible metal is melted, whereby the damper is closed and the supply of gas is shut off. The chamber is fitted with bars and hooks on which to suspend the articles of clothing to be disinfected. When the stove is first lighted, the gas is admitted to the burners direct through a short circuit pipe, without passing through the regulator, but when the mercury in the latter has risen high enough to reach the slit, this pipe is closed by a trap, so as to make the gas pass through the regulator, which is furnished with an adjusting screw, so that it can be worked at a higher or lower temperature, as required.

A much more simply-constructed apparatus than either the Washington Lyon or any of the preceding, is one invented by Van Overbeck de Meyer, in which the steam is generated in a jacket-boiler which surrounds the chamber, into which it is admitted through an opening at the top, but as it is not under high pressure, less strength of material is necessary, and safety valves or pressure gauges are not required. Owing to its lighter specific gravity, the steam does not mix with the cold air in the chamber, but displaces it, and drives it out through an opening at the bottom. Although the temperature does not exceed 212 deg. F., it is said that the penetrating power is only a little less rapid than it is in other forms of apparatus where higher temperature and pressure are employed. On account of its cheapness, this apparatus seems to be specially adapted for small districts.

### Plague at Bombay.

By a Nursing Sister.

Since my arrival in India for "plague" duty I have written an account of our work from Bombay, Poona, and Karachee, at all of which places I have had the pleasure of nursing, and now that I have nearly completed my year's service, I hope to give a short *résumé* of our work.

At present I am nursing in the Arthur Road Hospital, and I like it immensely. The wards are excellently built, and are quite the most airy and pleasant of any I have seen. The floors are of stone, which can so easily be washed and kept clean, and the wards themselves may be described as being built in two parts. The inner one is just a light enclosure of matting in wooden frames, with large open spaces top and bottom, and the whole is sheltered by a large, high, brick ceiling, supported by a light iron framework, and there is a nice stone verandah all round. As regards ventilation, nothing could be better.

Arthur Road Hospital has had the reputation of being one of the hardest-worked hospitals in India, and so, no doubt, it must have been last winter, when "plague" was at its height, but since I have been working here during part of the summer the work has not been hard, and we have not been too busy. There have been only three English sisters on duty, two in the daytime and one at night, and I think the day hours have been very well arranged. We have only taken duty every alternate morning and afternoon. For instance, the day sister would go on duty at half-past one p.m., and would be relieved by the night sister at eight o'clock. Then she would be on again next morning at seven a.m. until half-past one p.m., when the other day sister would come on and take similar duty, so that every alternate day we are off duty for twenty-four hours. This can, of course, only be done while the work is light, and we have been fortunate enough to have it so during this intensely hot weather. As regards night duty, the hours are too long for "plague" work. Each sister goes on after every fortnight and has a week's night duty, eleven hours at a time, and as we find it too hot to sleep much in the daytime we are extremely glad when our week of night work is over. However, it would be difficult to arrange it in any other way. Unfortunately we live a long way from the hospital, and so some of our time is spent driving backwards and forwards, and the sister on duty is obliged to have her breakfast sent down to her, and also her afternoon tea, which she takes in a small bungalow provided for the purpose. I have no doubt, though, that the drive does us good.

We are working under a Parsee doctor, who is extremely quick, methodical, and simple in his treatment, which consists largely in giving stimulants, &c. The local treatment is ice, which is undoubtedly good, but, oh, it is so difficult to prevent the patients lying in wet beds, which is very uncomfortable, even in this climate, and would be utterly impracticable at home. The constant application of ice has proved to be of great value, and it is wonderful how effectually the bubo subsides. We have had a number of our patients inoculated with the Italian serum, with some good results when taken at quite an early stage; indeed, the effect on the temperature, pulse, and respiration is most marked. We are now trying yet another remedy, viz., vapour and cold baths, but I cannot say much yet for the results of this treatment. It is certainly soothing and clean.

There are about three plague hospitals now open in Bombay, and I must refer to the sad tragedy lately enacted in one of them, viz., the "Mahratta Hospital," which is very popular amongst a certain caste of natives, as they are allowed to choose any treatment they like—I mean English or native—and have greater freedom and licence allowed them than at other hospitals. Everything had worked very happily until quite recently, when within three weeks the head native doctor, his three assistants, two dispensers, and three wardboys have all had "plague," and have all succumbed to it. Thus, at one fell swoop, the whole medical staff have fallen victims to this awful disease, and not one of them has survived. It has caused a great gloom to fall over this popular hospital, and the sisters have felt very sad at seeing each one of these, their fellow-workers, fall ill and die in turn. Truly it is awful to contemplate. Several doctors have been down to examine the wards, &c., and although improvements have been made as regards ventilation, there is nothing definite to show what has caused this infection amongst the hospital staff, except that dead rats have been found in the flooring of the office, where these poor men used to work. I thought of those words of Carlyle's, when hearing of their fate, "Beautiful it is to see and understand that no worth known, or unknown, can die even in this earth, for the working of the good and brave endures literally for ever, and cannot die."

The weather at present is most trying; the monsoon is over and the heat is extreme. We are now having the last of it however, and may soon expect lovely cool days, and quite envy those who intend spending the winter in India. Several of the nurses have decided to sign for another year's service, and in some cases they are wise to stay, but, at the same time, I think that had we been allowed to sign for a further term of six months, instead of a year, many more would have done so. There is no increase of pay offered to nurses who stay another year, except for those who work in Bombay, and as it is said to be an expensive town, the sisters working here are to get an additional Rs.15 a month. Nurses who are not doing anything special at home, or are not on the look-out for good hospital appointments, do well to stay here as long as possible. The pay is good, and the life perhaps more pleasant in some ways than they may find it at home; but otherwise two years spent away from hospital we know cannot improve one's knowledge on nursing matters, especially as things are done in a somewhat crude manner abroad.

The lady superintendents of Bombay and Poona receive Rs. 25 extra per month. Their duties are merely nominal; they are supposed to look after the housekeeping for the sisters, but as this appears to be done by contract with the butler it is not an arduous undertaking; they have no regular nursing duties, but are supposed to take temporary work when necessary, or should any sister fall ill, but as this is rare, I think, on the whole, this is a fairly easy appointment. In other places the superintendents do the nursing, order the diets for the patients, look after, and are sometimes held responsible for, the hospital property.

Poona has been until quite lately free from plague, and owing to the charming climate Government has arranged that those nurses who are at present unemployed may live there, which is extremely nice for them.

Calcutta has now been declared free from "plague," and I feel sure everyone must be pleased to hear this, for had this fatal disease once taken hold there, they say it would have been most serious, as it is such a crowded city. Bangalore, unfortunately, is suffering much at present, and some of our sisters have been sent there. I fear it is quite uncertain yet how many towns are likely to escape until the cold weather sets in, which is the time we have to dread.

I hear on good authority that the Burmah Municipality are behaving most generously to the Sisters, and are paying them Rs.250 a month each, but I do not know if they also provide free quarters, as we have here. In any case, however, it is very good pay.

The natives of Bombay appear to be most friendly, and our patients have the greatest confidence in us, and are sure that we have their welfare at heart; and in many cases they will take food and medicine from the sisters when they refuse it from everyone else. The Ayahs and ward-boys have improved wonderfully, and are quite clever, and most anxious to learn English.

On the whole, I think the nurses have kept very well in India; this is, doubtless, owing to their regular hours, plain food, and employment. India is a very relaxing place, and, unless you have any regular occupation, you are apt to become lazy, and by degrees you give up taking exercise and devote yourself to two or three hours' sleep in the afternoon, followed by a drive in the evening. Not to say that this is not an alluring picture, instead of having to drive in a hot sun to a "plague" hospital, and then to stay on duty in the most trying temperature with your collar limp and yourself decidedly moist! At the same time, you very soon forget—or rather get used to—your own condition when you see all the suffering around you, and the time passes away very quickly.

I think most of the nurses will agree with me in saying we have had a happy time nursing in India. In spite of the trying climate and hard work that has sometimes fallen to our lot, things have been arranged as comfortably as possible for our welfare. And if there has been an occasional grumble, why! is there any place in this world where we may expect to find perfection? Personally, I shall look back with pleasure to the year I have spent "plague" nursing, and I sincerely trust that our efforts may be crowned with success.

### Christmas Entertainments.

### THE LONDON HOSPITAL.

CHRISTMAS is always a very festive time at The London, sisters, nurses, medical staff, and students heartily combining to make the season a happy one for the patients. This year the "variety entertainments" got up by the resident staff and students, under the direction of Mr. Barnard, surgical registrar, proved particularly excellent, and were an unqualified success. The usual teas in all the wards, followed by entertainments by the various "troupes," took place this year on Christmas Eve. The children's Christmas trees followed on Wednesday. Year by year the number of friends anxious to come to this gathering of past and present "Londoners" has increased, until "Queen" and "Beatrice" wards have proved quite unequal to the strain upon their space. A change in the programme was therefore made on the present occasion, and the visitors on arrival found tea prepared for them in the new temporary wards, which have been erected in the garden for the accommodation of patients during the structural alterations soon to begin. The children had the delights of their trees to themselves before the crowds of guests were admitted into the two wards. Here all sorts of wonderful devices had kept the children amused all day; quaint decorations adorned all available spaces, and the trees, sent as usual by Mr. Leopold de Rothschild, surpassed those of other years in splendour and brilliancy. All the wards, as usual, were "on show," looking particularly bright and gay with coloured and shaded lamps, and an ample supply of cut flowers and plants. The "lobbies" of the older wards were prettily arranged with flowers and ivy, with a result quite as effective as and far more sensible than in the days when heavier wreathings of holly and evergreens were the customary form of Christmas decoration.

### EAST LONDON HOSPITAL FOR CHILDREN, SHADWELL.

SHARP is the contrast between the wretchedness and squalor of Shadwell and the bright and cheerful interior of the East London Hospital for Children, accentuated last week when Father Christmas had been busy at his pleasure-giving work, and toys were everywhere, and entertainments in full swing. Some people—people who have not looked with truly seeing eyes into hospital wards at Christmas time—say that these festivities are bad for the patients. These "conscientious objectors" would surely change their minds could they have seen the beaming little faces grouped on mattresses and blankets round the two tall trees, laden with pretty gifts, which graced the out-patient waiting-room, and watched the children's eager eyes following the Punch and Judy drama, an apparently inexhaustible amusement. The children at Shadwell had their Christmas tea party in the wards before the entertainment. Sisters and nurses, as usual, had made their wards look charming with flowers, their yearly custom being to fetch these from Covent Garden in the dark and early hours of the morning. The result in trailing smilax and yellow jonquils made the tables and window sills a feast to the eye. The children, too, thoroughly appreciated the glory of their scarlet flannel and dainty white garments, and placidly received the admiration of their visitors.

### LONDON TEMPERANCE HOSPITAL.

THE Christmas treat to the Temperance Hospital patients came off last Friday. There was a grand tree for the small patients lighted with electric light and covered with pretty gifts and glistening things. A concert was provided for the older patients' amusement, the stage being at one end of the unused ward and the tree at the other, and here all the patients well enough to enjoy the fun were brought. To judge by the general aspect of things, the evening was very greatly enjoyed by one and all, and the patients returned to their respective wards laden with many an acceptable present. Truly Santa Claus finds plenty of occupation at Christmas in our London hospitals! Miss Lucas had a busy time seeing to everyone's comfort and happiness; it was a great pleasure to her and to her guests to see Miss Orme in the midst of her old friends and the scenes of her past work. The wards, which are perhaps the most charmingly appointed of any in London, looked very bright and cheerful with their flowers and softly-shaded electric lights. Here, as almost everywhere, modern surgical science has pronounced against the old fashion of decoration, but to most people's taste the colour effects of lights, and fresh, sweet flowers are infinitely more to be admired.

### POPLAR ACCIDENT HOSPITAL, BLACKWALL, E.

CHRISTMAS entertainments at Poplar have been divided between two days. Last Friday, "Friends from the London Hospital" gave a dramatic entertainment in one of the wards, while special amusement in the shape of a Christmas tree was provided for the children on the Wednesday following. The "Friends" responsible for the first night's performance were the kind and energetic providers of similar entertainments at their own hospital, at the Metropolitan Hospital, and at Moorfields, and most warmly must they be congratulated on the immense success of their efforts. In addition to the "Sketches" of the "Crocks," the "Pierrots," and the "Jonathan Johnnies," which were repeated with as much spirit as though done for the first time, and with many a particular joke added for the special benefit of the Poplar audience, greeted with shouts of laughter, these indefatigable actors gave the always popular "Box and Cox," and an excellent "Variety Entertainment." Such songs as "Jujah," with an extra topical verse,

"Tatcho," "The Twins," &c., met with vigorous applause, and favourite choruses were joined in with great energy by the entire audience. The "Pierrots" performance concluded the entertainment, and they wound up with several well-worn and popular plantation songs, "The Old Banjo," and other favourites.

THE NATIONAL HOSPITAL FOR THE PARALYSED AND EPILEPTIC.

THE wards and chapel of this hospital were decorated for Christmas, and early on the morning of the day the nurses sang carols, after which breakfast was served to the patients, who each received a present. A service was then held in the chapel, and dinner brought turkeys, beef, plum puddings, and dessert to the fore, and the children had a tree in the Duchess of Albany ward. A succession of ward teas will take place during the week, and the holiday-making will end on the 7th inst., when a concert will be given to the nurses and their friends.

THE HOSPITAL FOR SICK CHILDREN, GREAT ORMOND STREET.

No pains were spared by the promoters of the annual treat to the little out-patients of the Hospital for Sick Children to render it a memorable occasion in the lives of the 200 little guests privileged by twelve months' attendance at the hospital to be present. The sisters of the north out-patient and the south out-patient departments, assisted by willing and efficient helpers, amongst whom the house surgeon distinguished himself, transformed the whole place into a fairyland of coloured lights, festooned draperies, and greenery. Red was the prevalent colour in the visitors' tea room, and another guest-room was decorated in green and pale blue. A Santa Claus beamed a seasonable greeting in the room where the youngsters feasted heartily of innumerable good things—for the most part gifts of friends—and a long entertainment, beginning with a Punch and Judy Show, continuing with the scene "The Mad Hatter's Tea Party" from "Alice in Wonderland" (happily chosen, for this hospital has now an "Alice in Wonderland Cot" in memory of the writer), and ending with the distribution by Dr. Colman of 200 packets of clothing, each packed by the kind nurses with reference to the needs of its future owner. The children were then permitted to behold the glories of an illuminated Christmas tree in batches of 20 at a time, and then, rich in the possession of a share of its coveted adornments, they were sent happily home to dream of the delights from which otherwise they could not have torn themselves away.

UNIVERSITY COLLEGE HOSPITAL.

ON Thursday, December 29th, the patients and nurses' annual entertainment took place at this hospital. The wards and corridors were prettily decorated with wreaths of evergreens, flags, and draperies. The tables were laden with plants, fruit crackers, and parcels of gifts. In ward 12 Mrs. Walter Scott arranged a concert, and the concert room downstairs was filled with patients, nurses, guests, &c. Nurses Violet Vallings, Edith Denny, Agnes Pitman, Nellie Wrighton, and Messrs. Clover, Etlinger, Pitman, and Benham sang two of Scott Gatty's plantation songs very tastefully. Mr. Harry Davenport won great applause by his rendering of "Lucky Jim." Miss Magdeline Blathwayt played a "Romance" by Fischer on the violoncello, and "Hejri Kati," by Hubay, on the violin with much sympathy; whilst Dr. Frederick T. Roberts sang most charmingly "The Anchor's Weighed" and "Sally in our Alley." The second part of the programme was a short farce entitled "The Area Belle."

WEST HAM HOSPITAL.

ON Christmas Eve the wards of West Ham Hospital were made bright and cheerful by decorations, a large Christmas tree, and the presence of visitors. The tree was laden with gifts, and amongst the many useful articles were toys and games to while away the long hours of sickness and convalescence. There is no doubt about the delight in the patients in hospital at Christmas time at the merrymaking, however much opinions of other folk may be divided upon the subject of its advisability. At West Ham, doctors, nurses, and patients agree upon the point, and everyone was made as cheerful as possible. The secretary, doctors, matron, and nurses did the honour of the entertainments with cordiality and gaiety, and invit d the guests to come again.

THE ROYAL WEST OF ENGLAND SANATORIUM, WESTON-SUPER-MARE.

THE festivities of this sanatorium on Christmas Eve were most successful. The dining hall and reading room were charmingly decorated. In the former a feast of good things to eat was provided at five o'clock, and in the other a programme of excellent music was afterwards much appreciated. Nurses Florence, Jean, Lilly, and Katherine, whose music and singing often afford much pleasure in the evenings, contributed a fair share to the evening's enjoyment, in which Dr. Roxburgh, Dr. Knox, Mrs. Thomas, Miss Charter, and Mr. and Mrs. Comfort took part. The entertainment was arranged by the lady superintendent and her nurses, who had the satisfaction of knowing that the evening was a delightful one to their guests.

CHILDREN'S DAY AT ST. THOMAS'S HOSPITAL.

IT was "Children's Day" at St. Thomas's Hospital last Saturday. Victoria Ward was very much *en fête* indeed, and the children on the tip-top of pleasurable excitement. Tea, at a quarter to three, was the first item on the programme, to which the children from the other wards were invited, followed by a marionette show, while Sister Victoria was dispensing tea to her guests. Then Father Christmas made his welcome appearance, and the children were gathered in cots, and blankets, and in the nurses' arms, round the splendid tree, sparkling with electric light and laden with pretty things. It is one of the most touching things in the world, I think, to see the pleasure of hospital children in the simple Christmas joys provided for them, and if the children's enjoyment is great it is certain that their elders find even greater pleasure in witnessing it.

Sister Victoria had a happy inspiration this Christmas, originating from her desire to get together all the dolls she wanted for her little patients. This was a "Doll Competition," with several classes of entries, one for students, another for students' womenfolk, and others for sisters and nurses and for students' "friends." Dozens of dolls resulted from this brilliant idea, which "caught on" with enthusiasm, especially amongst the students. The judging took place on Saturday, the task being undertaken by Miss Gordon and Mrs. Swann, with the help of two members of the visiting staff and two of the residents. Sister Victoria's prizes were baskets of flowers and boxes of bonbons, and these were received amid much applause by the successful competitors. Some of the dolls were beyond praise for cleverly thought out costumes and wonderfully well-finished as to detail.

In the students' class for the best-dressed dolls the prizes were carried off by Mr. Fry, Mr. Pern (a Pierrot), and Mr. Harrison, whose "Jovial Monk" earned much praise. A fourth and special prize was presented to Mr. Singer for a doll clothed entirely in surgical dressings—her frock of cyanide gauze, with petticoats of iodoform gauze, cap of wool ornamented with a star of "protective," sash of lint, and stockings and boots of strapping. The sisters of Mr. Harrison, Mr. Bourdas, and Mr. Patch were successful in the second class; Nurses Izod and Clarke, and a patient in the home carried off prizes in the third class. The dolls were drawn for and triumphantly appropriated by their proud possessors.

But the first prize of all was awarded to Mr. Williams for a "case" complete—bed, patient, and every imaginable appliance, all in miniature. The white-painted bedstead with movable sides was made up with the most minute attention to detail as a fracture bed (the bedclothes, &c., were made by one of the ward sisters); the small patient, "Sally Cilate," aged six, suffering from a variety of injuries, but chiefly from a fractured femur, put up with P.P.S., and long outside, and beautifully-applied bandages, even to an extension, with weight, marked 4 lb. "Sally" was also provided with a tiny bed table; on it a wee basin with clinical thermometer, specially blown (like the labelled medicine bottle) in the laboratory, and a bronchitis kettle and stand to match. Temperature chart, carefully worked out, temperature varying wildly between 96 deg. and 107 deg.; case board, an excellent imitation of the "real thing"; and a complete history of the case hung beside the patient. The notes are so amusing that they may well be given in full for the edification of those who were not there to see for themselves (they were written by Mr. H. G. Pinches):—

Medical Officer, Mr. —— Ward, Victoria, No. ½....
Clerk or Dresser, Mr. Williams.
Nature of Case (to be entered by the Registrar)..................

Name, Sally Cilate. Age 6. Sex, Female.

Admitted, Dec. 20, 1898.

Discharged............ Residence, Frying Pan Alley.

HISTORY.

*Notes by Medical Officer or Registrar.*

*Report by Clerk or Dresser.*

*F. H.*—Father died of drink. Mother doing time (Wormwood Scrubbs) Brother-in-law, who is a coal heaver, suffers from spasm of the epiglottis. Pat. has 12 brothers and sisters who of which are at Barnum's (Freak Department).

*P. H.*—The child was born on a Friday and is the 13th in the family. No other serious illness. Pat. was vaccinated on the L. arm when 3 months old, since when it has never been the same child. Pat. often suffers from the hump, which is worse after it has been smacked. Pat. spent her last holiday at Margate, where while paddling she caught a cold, 2 crabs, and a flat fish. No history of alcohol.

HISTORY OF ACCIDENT.—The child whilst returning from the "Bee and Gum-pot" with the morning beer in a jug without a handle, was run into and knocked down by a steam-roller in the Westminster Bridge Road. The engine driver in order to avert an accident shut off steam, and put on the patent vacuum brake, back pedalling at the same time, but this had not the slightest effect in reducing the speed of the engine, which did not come to a standstill until it struck the child, the front wheels remaining stationary on Pat.'s legs. There were no rubber tyres on the front wheels; in the excitement of the moment the child dropped the beer jug, which however only broke into 8 pieces, but is quite unfit for further use except as a flower vase.

The child was picked up by Constable 98·4 who violently blew his whistle for assistance, and drank the small quantity of beer which was left in the jug. After most of the Lambeth Police Force had arrived, and all had produced note books, in which they entered the case at great length, the child was brought to the hospital by 8 constables, 3 inspectors, and an ambulance; the wheels of the latter had refused to go round, so it was carried by several additional policemen.

Will the H S. kindly see that the dresser writes his notes.

ON ADMISSION a pale looking child, with a flushed face and gold hair (4/6 the large bottle) suffering from fractured femur, the fracture is situated at the junction of the lower 10/11 with the upper 7/19. Transverse in direction, the upper fragment looked a little outwards, backwards, forwards, and inwards. Large coarse crepitations and Rales could be felt. The R. Tibia and Fibula are also fractured, but this was not discovered till later as there was a good deal of contusion. Knee jerks were absent, but the abdomen moved well on respiration. The child repeatedly called for a drink (the father was a drunkard) and seemed to be in some pain, but this was probably due to the injury. There was also a small scalp wound about 5/10 inch long, situated in the region of the ant. sup. angle of the L. parietal bone. The L eye of the Pat. was somewhat contused, and the child says that a horse or one of the Police Force trod on it. As the eye cannot be opened the pupil does not react to light or accommodation. Nothing abnormal in the disc.

PROGRESS OF CASE.—DIET AND TREATMENT.

Dec. 25.—Dr. H—— examined Pat. and found over the base of left lung behind there is an area of dulness, about the size of a *Rugby football.*

Breath sounds absent. Numerous *Ronki* are seen of a bubbling character over the epigastrium, these Ronki are only visible during dighilition. Heart sounds normal on both apices. But the diastolic murmur is only audible when the child is screaming. During the convulsions the excretion of urea was increased as seen by the hyper-pyrexia. Abdomen resonant all over the p.p.s. Pulse was imperceptible, but otherwise normal. Dr. H—— was of opinion that the child's condition was critical.

Dec. 26.

? Barcelona Nut.

No Rhonki to-day.

? Systolic murmur at Rt. Apex

Tender mass in posterior mediasternum.

Abdomen dull all over. Pulse full and bounding. I would advise a Quencke's puncture or a course of Weir Mitchell treatment.

### Minor Appointments.

ROYAL HOSPITAL FOR CHILDREN, ST. MICHAEL'S HILL, BRISTOL.—Miss Marian Thompson was appointed Sister-Housekeeper here on December 28th. She was trained at the General Hospital, Birmingham, and has been nurse-matron of Sir Titus Salt's Hospital, Shipley, and night superintendent, the Infirmary, Burton-on-Trent.

BELFAST NURSES HOME, FREDERICK STREET, BELFAST.—Miss Lucy Murray has been appointed Home Sister here. She was trained at the Leeds General Infirmary, and she has been charge nurse at the Grantham Hospital and at the General Infirmary, Aylesbury; and night superintendent of the Clayton Hospital, Wakefield.

THE PARK HOSPITAL, HITHER GREEN, S.E.—Miss Mary Ann Houghton, who was trained at the Poplar and Stepney Sick Asylum and at the Orthopædic Hospital, Great Portland Street, was appointed Charge Nurse here on December 30th, 1898.

ST. SAVIOUR'S UNION INFIRMARY, S.E.—Miss Margaret Urquhart, who was trained at Charing Cross Hospital, has been appointed Sister here. Miss Urquhart was previously sister at the Victoria Hospital, Broadstairs.

### Where to Go.

DOWDESWELL GALLERIES.—An interesting collection of pencil drawings by E. Borough Johnson, R.B.A., and of water colours by Montague Smyth, R.B.A., representing scenes in East Anglia, will be on view at the above galleries, 160, New Bond Street, next week.

THE NATIONAL ANIMALS HOSPITAL FUND.—A dramatic and musical performance will be given on the 23rd inst., at 8 o'clock, at St. George's Hall, Langham Place. It is under the patronage of the Earl of Sandwich, and the performers are aristocratic amateurs.

### On "Christmas in Hospital."

THERE has been discussion in the papers lately on the subject of Christmas Entertainments in Hospitals, some writers expressing the opinion that such festivities are harmful to the patients. But there are two sides to this as to every other question, and most sensible people, with any practical qualifications for pronouncing an opinion, incline to the view that the attempt to carry something of the joyousness of Christmas-tide into the wards of our hospitals is largely productive of good, and that in more ways than one. No one can better estimate the net result of these amusements, so far as the patients are concerned, than the ward sisters in those institutions where entertainments are the rule at this season, and from special enquiries made it is clear that the weight of their experience goes to show that the patients are the better, physically and mentally, for the pleasure thus brought into their lives. Great care is taken nowadays to ensure that evil results shall not follow. Asepticism demands that dust-collecting decorations shall no longer be allowed, and their absence has been conspicuous everywhere this year, though with no diminution of effect, thanks to abundance of flowers, coloured lights, and other surgically harmless devices. For instance, on the "Tree" day at St. Thomas's it was noticeable that the tree was far away from any possible contact with surgical tables and dressings and cots, while the ward was as bright and festive with electric light and flowers as ever in old days of holly garlands and unstinted drapery. Here also it was good to learn afterwards that, so far from harm accruing to the children none were the worse and many the better by a great deal of happiness for their Christmas treats. How joyless are some of the lives thus brightened perhaps only hospital workers can appreciate, and surely the spirit of peace and goodwill, so strongly evident at Christmas time in hospital, is not without its educative effect on those who come under its influence.

And, again, how humanising is the effect upon hospital workers themselves, men and women! Christmas, as a busy sister said the other day, is something to look forward to all the year through—a bright oasis in the annual routine. One has only to watch the genuine satisfaction of doctors and students, sisters and nurses, in the pleasure of their patients to mark how the endeavour to spread happiness brings the truest happiness itself; to see the children turning for never-failing sympathy to their special friend in nurse and "doctor," and the ungrudging way in which the latter give of their time and labour, to realise that the keeping of Christmas in hospital does much to bring out all that is best in the men and women within its walls, and is thus a potent factor for good to all.

### The Pension Fund Nurses.

### MISS BURNS'S WEDDING GIFT.

WE publish below a further list of nurses who are contributing to the address to be presented to Miss Burns, daughter of the late chairman of the Pension Fund, on her marriage. The contributions, which are limited to 6d., are received by the Editor, The Lodge, Porchester Square, W. All names will be forwarded with the address, and this will be on show when completed: S. Brookes, A. Turner, Brearey, E. Parsons, L. S. King, E. Graham, A. L. Spreat, M. E. Spreat, J. Girrard, Eggleston, E. Burrows, M. Frost, E. Lyons, A. M. Kelly, Winter, Loyd, Fisher, E. A. Dann, L. Harding, M. Perceval, C. S. Phillips, E. J. Dennington, E. Howe, M. Church, J. C. Sherlock, Chawner, J. E. Brown, M. Byrne, A. M. Lane, J. Walker, G. E. Jones, S. A. Henwood, E E. Wilson, Policy No. 498, Policy No. 3,749, S. A. Boher, E. Lews, A. Middleton, A. Garratt, E. M. Williams, A. Michie, E. Snell, E. Cooper, A. Cottle, S. J. O'Leary, M. Byers, E. Hatton, L. White.

### Queen Victoria's Jubilee Institute for Nurses.

### NEW YEAR APPOINTMENTS AS "QUEEN'S NURSES."

THE Queen has approved the appointment of the following nurses to Queen Victoria's Jubilee Institute for Nurses, to date January 1st, 1899:—

ENGLAND.—Emily Miller, Frederica Roberts, Emily Eliza Abraham, Mary Beatrice Traiforos, Agnes Anne Corrie, Ada Rebecca Griffin, Helena Ralph, Lillie Gorrill Turner, Christine A. Croome, and Emily Jane Davies, all working in London; Mary Ann Harrod, and Clara Elizabeth Craddock, East London; Mary E. Ragg, London; Clara Clews, Hanley; Jessie Whan, Norton; Christina Mackay Connon, Winslow; Florence Mary Youlden, Southampton; Alice Westaway, Rawtenstall; Mary Wilson Charters, Ryde; Amelie Julie Allen, Banbury; Annie Trousdale, and Ellen Mary Wass, Cheltenham; Charlotte Evelyn Jex-Blake, and Amelia Sarah Holbrook, Gosport; Lily Gertrude Steedman, Coventry; Eliza Reed Kelsey, Chesham; Clarice Hinings, Florence Annie Smith, Jessie Thorn Wright, Elizabeth White Gray, and Elisa Brash MacArthur, Manchester; Margaret Scott, St. Neots; Mary Ann Cracknell, Barton-under-Needwood; Maud Hartwell Lane, Blackburn; Minnie Granville Smith, Quedgeley; Ethel Louisa Prout, Aldershot; Isabel Douglas Wright, Isabella Strickland, Elizabeth Fleming, Margaretta Louisa Colvin, Jane Hughes, Ada Willacy, Elizabeth Brayden, and Beatrice Mary Exton, Liverpool; Ellen Elizabeth Havers, Burgess Hill; Charlotte Scarfe, Truro; Catharine Mary Walker, Marple Bridge; Mary Elizabeth Jeffries, Mary Stewart Holland, Florence Gertrude Hughes, and Kate Taylor, Consett; Alice Harcourt, Hertford; Edith Chamberlain, Reading; Maud Stone, Bolton; Sarah A. A. Milton, Leeds; Adeline Haynes, Barnstaple; Mary Louisa Culwich, Longtown; Mary Gladwin, Wisbech; Ellen Munday, Gateshead; Marion Pile, Gloucester; Edith Shooter, Annie O'Neill, and Annie Walker, Northampton; Katharine Harbord, Brighton; Grace L. Brown, Darlington; Hilda Mary Good, Cleator; Mary C. Browne, Windsor; Constance Catharine Wells, Harpenden.

WALES.—Margaret Ellen Lewis, working at Cardiff; Ellen Georgina Mealey, Llantrissant; Elizabeth Ann Schofield, Pontyclun; Charlotte Amelia Williams, Ruthin; Henrietta Sykes, Dungleddy; Annie Evans, Portmadoc; Ethel L. Dean, Bangor.

SCOTLAND.—Margaret McFayden, Rachael Booth, Mina Douglas, and Margaret Barnet, working at Edinburgh; Annie Willox, Elizabeth Calderhead, Jessie Mill, Margaret MacLennan, Jenny McMartin, and Elizabeth Melville, Glasgow; Catharine Gordon and Margaret Helen Swan, Dundee; Annabella McNeillie, Hawick; Helen Jane Smith, Paisley; Helen Frances Gentle, Innerleithen; Christina Cameron, Glencoe; Rachel Elizabeth Morrow, Newburgh; Jessie D. K. Balfour and Katharine Margaret Mackenzie, Elgin; Bessie Knox Crawford, Hamilton; Helen Noble, Port Glasgow; Jessie Mackay, Clachan and Killear; Maggie Melville Rae, Wick; Elizabeth Adair Mill and Mary Gourlay, Barrhead; Margaret Dick, Bellshill; Eliza Harvey, Rothesay; Gertrude Eleanor Sothern, Ratho.

IRELAND.—Susan Reid Shaw, Jane Jackson, Hannah Charley, and Rose McKeown, working at Dublin; Mary Louisa Wilkinson, Armagh; Ethel Christina Hazleton, Ballinamallard; Sarah Agnes Stack, Cappoquin.

### "THE HOSPITAL" CONVALESCENT FUND.

IT is with much pleasure that we acknowledge 3s. from Miss E. M. Williams; 6s. from Nurse Lucy White; and 5s. from Nurse Cecil, for the above fund, and hope that this, our first fruits of the new year, may be an acceptable example for others to follow. We want this most useful fund to be more widely helpful, and it can only be so through the united efforts of our kind readers and friends.

### THE VICTORIA COMMEMORATION CLUB.

THE secretary of the above club begs us to announce that in response to many urgent requests it has been decided for the present to abolish the entrance fee for nurse members of the club until further notice. The subscription remains at £1 1s. per annum.

### For Reading to the Sick.

OUR PERPLEXITIES.

**Verses:**

Some groping at Faith's door in misty doubt,
And worn by conflict, from the Truth shut out.
To all these woeful souls a Christmas morn
Brings but new grief and weariness forlorn.
Then bid them gaze t'wards Calvary's dark hill,
Where He, our sacrifice, bleeds for us still—
Sinless, compassionate—for me, for you;
Yea, mortal anguish to the full He knew.
Faint hearts! Christ's message wings not to the glad.
He calls the blind, the lame, the sick, the sad,
The Christmas of the sorrowful for sure,
Within His own short span did He endure.
When here His latest wintry days were spent,
He wrestled sore in prayer, and silent went
Out to the desert, sorrow led, where dim
The future loomed, and Death encompassed Him.
His hours as holy stairs led up to God—
Steps that His aching, bruised feet slow trod.
Dwell ye on this, ye that repine and fret,
That He may lift and walk beside you yet.

—*Lady Lindsay.*

**Reading.**

What is the divine life-plan? What are we to do with our cares?

Everything that threatens to give us anxiety is to be taken at once to God. Nothing is too great to carry to Him. Does not He bear up all worlds? Does not He rule over all the affairs of the universe? Is there any matter in our life, how great soever it may seem to us, too hard for Him to manage? Is any perplexity too sore for Him to resolve? Is any human despair too dark for Him to illumine with hope? Is there any tangle or confusion out of which He cannot extricate us? Or is anything too small to bring to Him? Is He not our Father, and is He not interested in whatever concerns us? There is not one of the countless things that fly like specks of dust all through our daily life, tending to vex us, that we may not take to God. And this is the cure which the Scriptures prescribe for care. The divine philosophy of living says: Be anxious for nothing, but make everything known to God; in everything, by prayer and thanksgiving, let your requests be made known unto God. . . . It is not enough to kneel down and make a prayer, nor is it enough to pray about the particular matter that worries us, asking for help or deliverance. Only the most simple-hearted definiteness in prayer will meet the need. We must bring the very perplexity itself and put it out of our hands into God's, that He may work it out for us. . . . And having done this we are to cease to worry. We have given the perplexity to God. We have asked Him to think for us, plan for us, and take the ordering of the affair into His own hands. It is our matter, therefore, no longer, but His. . . . Then having taken it to Him and put it into His Hands, we are to leave it with Him; having gotten it off our own shoulder upon His, we are to allow it to remain there. But it is just at this point that most of us fail. We tell God about our worries, and then go on worrying still, as if we had never gone to Him at all, or as if He had refused to help us. We pray about our cares, but do not cast them off. We make supplications, but do not unlade our burdens. Praying does no good. It makes us no more contented or submissive, or patient, or peaceful. We do not get the worries out of our own hands at all. This is the vital point in the whole matter. Or perhaps we do not cast the burden upon God while we are praying, and feel for the moment a strange sense of joy in the soul. We rise and go a few steps as light-hearted as an angel. We have given God our cares to keep. But in a little while we have gathered up all the old burdens and anxieties again, and have them once more on our own shoulder, and we go on bowing under them, fretting and worrying as before.—*J. R. Miller.*

### Appointments.

Lincoln County Hospital.—Miss Mary E. Ray was elected Matron of this hospital on December 29th. She was trained at King's College Hospital, London, and since March, 1896, has been assistant superintendent of nurses at the General Infirmary, Leeds.

### Notes and Queries.

**The contents of the Editor's Letter-box have now reached such unwieldy proportions that it has become necessary to establish a hard and fast rule regarding Answers to Correspondents. In future, all questions requiring replies will continue to be answered in this column without any fee. If an answer is required by letter, a fee of half-a-crown must be enclosed with the note containing the enquiry. We are always pleased to help our numerous correspondents to the fullest extent, and we can trust them to sympathise in the overwhelming amount of writing which makes the new rules a necessity.**

**Every communication must be accompanied by the writer's name and address, otherwise it will receive no attention.**

*Midwives.*

(147) "Inquirer" would like to know whether a midwife trained at the Queen Charlotte's Hospital, and holding the certificate from that hospital, will be qualified to go on the register when State registration is accomplished, or will she be compelled to hold the L.O.S. certificate?

It is quite impossible to predict what regulations will be in force when State registration of midwives is a fact.

*Books for Preliminary Scientific Examination.*

(148) Would you kindly advise me through The Hospital what books you recommend for the Preliminary Scientific Examination, London University? Books that would give a good sound knowledge of the subjects without being very expensive.—*R. H. Williams.*

The University Correspondence College text books are cheap, sufficient for a pass, and written up to the syllabus. It is better, however, though more costly, to read Deschanel's Physics, Huxley and Martin's Botany, Marshall and Hurst's Zoology, and Dr. Luff's "Manual of Chemistry," with frequent reference to larger works.

*Nursing Old Age.*

(149) Can you tell me whether there is any book on the care and nursing of old age?—*E. M. T.*

"Nursing Old Age," by Mrs. Truman, published by the Roxburgh Press.

*Work in America.*

(150) Can you tell me if a well-trained nurse is likely to get work in America, and if so, what part of the country?—*E. E. J.*

Could you kindly tell me how I can obtain work as private nurse in New York? Am very anxious to go, but would like to know if it is possible to get a post before leaving England. Are there any agencies or institutions to which I could apply?—*M. B.*

An English nurse has much difficulty in getting a footing in America. She must have sufficient capital at first to maintain her in a suitable way until she gradually establishes herself. There are agencies or registries in the different large towns, and probably some would admit English trained nurses to membership. The advertisements in the *Trained Nurse*, published in New York (London agents, the Scientific Press), might help you, but the fact is that nurses are as plentiful in America as in England.

*Training School.*

(151) Can you name a hospital in the North-West or West of England which gives really good training where I could enter as free probationer at the age of 19 or 20? Good health; height 5 ft. 4 in.

The following hospitals of over 100 beds take probationers from 20 years of age: The Guest Hospital, Dudley; the Royal Halifax Infirmary; the Hereford General Infirmary; the Salford Royal Hospital, near Manchester; and the York County Hospital.

*A Nurse's Latin.*

(152) Will you let me know what book (cheap) can be recommended from which a nurse can learn the necessary amount of Latin to make her proficient in her duties, especially with regard to medical terms?—*W. S. W.*

A nurse may be quite proficient in her duties without any knowledge of Latin, but if she wishes to study Medical Latin she may read "Elements of Latin for Students in Medicine and Pharmacy." Crowther and Bios, publishers. The Scientific Press, London, would get it for you.

*Information Wanted.*

(153) I am the nurse-matron of an infectious hospital (12 beds) all scarlet fever. Our staff consists of a nurse, a male caretaker, and myself. Our medical officer expects us to do all the cooking, washing, and cleaning between us. Will you please say what staff we should have, independent of the above, to carry out this labour?

It is impossible to say what staff is necessary without an accurate knowledge of the requirements of the hospital, but it is not right to expect a nurse to do the work of a general servant and a laundress. When there are no patients in the hospital it would not be unreasonable to expect her to wait upon herself, but with the advent of even a single patient the nurses' duties are to nurse and nothing else.

*Skin Disease.*

(154). Can you tell me of a good hospital for skin diseases where a young lady would be received as patient? One at which Dr. Liveing attends would be preferred.—*Hospital.*

See "Burdett's Hospitals and Charities" (Scientific Press, London, price 5s.) for list of hospitals. Dr. Living is consulting physician to the Middlesex Hospital.

### Travel Notes.

By Our Travelling Correspondent.

### V.—NICE (*continued*).

St. Maurice is a suburb (decidedly uninteresting in itself) from which many lovely places are seen. It is reached by carriage or train, which latter runs each way every quarter of an hour. It is above the convent of St. Barthélemy that the Villa Arson, of which I send you a sketch, is situated. The train and the constant journeys of the hotel omnibus prevent one feeling at all shut out from the sea and the gaieties of Nice, and on Sundays all is made easy by the omnibus conveying residents to any church they wish to attend twice a day. My first view of this fascinating spot, when I made the accompanying sketch, was on a lovely spring morning. We had come over really to see the old Franciscan convent. The sun was brilliant, the sky the most vivid ultramarine, the hills showed every opalescent hue, whilst Mont Chauve still kept snow on his bald crown. We climbed up the heights to the left through some orchards, which were in full bloom, and reached an olive wood, where the trees of singular and weird growth made a natural frame, in which the lovely old villa stood out in all its stately quietness. Under my feet in the grassy terraces, on which the olives are planted, bloomed the purple iris, and a very beautiful flower hung in the rocky heights above, the name of which I am not a sufficiently learned botanist to know, but it is one of the loveliest I have ever seen, closely resembling our Japanese anemone, but of a vivid mauve colour. Since that morning I have made closer acquaintance with the Villa Arson, and love it dearly. Its terraced walks, its ilex shrubberies, its groves of palms, its old world statuary, and its ancient stone gateways make up a whole not easily matched. At its feet lies the monastery of St. Barthélemy, rearing its delicate cupola against the blue sky. This is said to have been built in imitation of the tower of the Palazzo Publico at Florence, and certainly closely resembles it. I can imagine nothing more delightful than to sit out on an early spring morning in this grand old garden, reading the last new novel, which one would readily put down to turn one's thoughts back to the romantic past and listen, as one gazes seawards, to the convent bell below ringing the Angelus, as it has done any time these three hundred years. The quaint surroundings transport one in thought far from the fret and worry of the nineteenth century, with its utilitarian business engagements, to the times of powder and patches, which seem to fit into one's ideas of the old villa; and again to the quiet convent below, where many a vexed and saddened life has sought to hide its grief in the silent cloister.

The Villa Arson from the Olive Woods.

### The Val Obscur.

A very easy morning's excursion for those in fair health is to the Val Obscur, a very singular gorge beyond St. Maurice. Take the tram to the terminus and then walk, but for a delicate person a carriage must be taken as far as it can go consistently with turning round. You drive up the dry bed of a torrent which is fed, I conclude, by the Vars It is not a fitting excursion for real invalids, because there must be walking and a certain amount (though in a very mild way) of gymnastic performances; it is called the Val Obscur on account of its great darkness, caused by the mighty rocks which tower 300 feet above one's head being so close together, in places only five and a half feet apart, that daylight is almost excluded. These rocks, which are as perpendicular as if sawn straight down, are richly clothed with maidenhair fern. The path, if such it can be called, along which you penetrate this darksome route is formed of stepping stones standing in the shallow torrent, and sometimes the jump from one to another is pretty long; there is absolutely no danger, the path is as safe as the hotel hall, but you can understand that it is an unsuitable expedition for any but the robust. By following this singular and romantic path for about half a-mile you come to the head of the valley, and must then turn up towards the left. A fairly-long and very beautiful walk, always ascending, will bring you out on the high road, distant from the beginning of the gorge about two and a half miles. I have never seen this round mentioned in the guide-books, but, remembering the time it took me to do it, I should estimate it to be about that. Having struck the road turn to the left, and you will reach Nice in half an hour. It is, of course, unnecessary to take this lengthy walk, lovely as it is. With a weakly person you might drive as far as practicable, and then follow the bed of the torrent to the head of the valley, and return the same way, without making the ascent at all.

[The first of this series of articles appeared on Dec. 10th, 1898.]

*Advertisements referring to travel will be found on page xxii. of our advertisements.*

### TRAVEL NOTES AND QUERIES.

St. Jean-de-Luz (Como).—Yes, there is a well-established English colony there, but it is not a gay place by any means. For that you must go to Biarritz. There are three good hotels at St. Jean, The Post, La France, and the Angleterre. Apartments are reasonable, but living not particularly cheap.

Algiers (Daisy).—Single, first-class, £9 12s. 4d.; return, £14 2s. 3d. Second, single, £6 14s. 3d.: return, £10 10s. This is via Newhaven and Dieppe. By Dover and Calais about £1 extra. Yes, it is more consistently warm than on the Riviera.

Douarnenez (Violin).—Douarnenez is on the sea coast department of Finistère. It would only be agreeable in the summer time. It is then much frequented by artists, but the situation is exposed to the full force of the Atlantic waves and winds.

Dieppe (Rosemary).—It is now a decidedly expensive place, and spoilt in consequence. The old picturesque town is almost swept away, and therefore not suitable for your purpose. Why not try Lisieux or Morlaix?

## Figures and Tables

**Figure f1:**